# Bedload transport in rivers, size matters but so does shape

**DOI:** 10.1038/s41598-020-79930-7

**Published:** 2021-01-12

**Authors:** Mathieu Cassel, Jérôme Lavé, Alain Recking, Jean-René Malavoi, Hervé Piégay

**Affiliations:** 1 University of Lyon, Lab. Environnement Ville Et Société, CNRS UMR 5600, Site ENS de Lyon, 15 Parvis René Descartes, BP 000F-69342, Lyon Cedex 07, France; 2 CRPG-CNRS, 15, rue Notre Dame des Pauvres, BP 20, 54500 Vandœuvre-lès-Nancy, France; 3 Inrae, UR ETNA, Domaine Universitaire, 2 rue de la papeterie, BP 76, 38402 Saint-Martin-d’Hères, France; 4 Département Concessions Eau Environnement Territoires, Electricité De France - EDF/DPIH, Le PRIMAT - 190 Rue Garibaldi, 69003 Lyon, France

**Keywords:** Geomorphology, Sedimentology

## Abstract

Bedload transport modelling in rivers takes into account the size and density of pebbles to estimate particle mobility, but does not formally consider particle shape. To address this issue and to compare the relative roles of the density and shape of particles, we performed original sediment transport experiments in an annular flume using molded artificial pebbles equipped with a radio frequency identification tracking system. The particles were designed with four distinct shapes and four different densities while having the same volume, and their speeds and distances traveled under constant hydraulic conditions were analyzed. The results show that particle shape has more influence than particle density on the resting time between particle displacement and the mean traveling distance. For all densities investigated, the particle shape systematically induced differences in travel distance that were strongly correlated (R^2^ = 0.94) with the Sneed and Folks shape index. Such shape influences, although often mentioned, are here quantified for the first time, demonstrating why and how they can be included in bedload transport models.

## Introduction

Sediment transport is a key process in fluvial geomorphology, being important for sustainable management of navigable channels, designing engineering projects, predicting morphological changes and associated hydraulic risks, interpreting sedimentary archives and restoring rivers^1^. It involves three phases of particle mobility: (1) entrainment^2–6^; (2) motion^7–9^; and (3) deposition^10,11^. Sediment transport at the particle scale is a stochastic phenomenon^7–9,12–14^, which mostly arises from the complex interactions between particle collisions and highly variable friction, drag, and lift forces due to fluid turbulence. Thus, for practical considerations, empirically calibrated sediment transport functions widely use the Shields stress number (τ*) to quantify the balance of the forces exerted on the channel bed particles, and the critical Shields number (τ*_c_), which is the threshold value necessary to set particles in motion, to determine the moments at which drag forces exceed stabilizing forces (τ* > τ*_c_) and particles can be entrained^15–21^. Such approaches have been used to estimate particle stabilizing forces from median pebble size and submerged density^16^. At the river reach scale, sediment transport estimates generally encapsulate a relation depending on the Shields stress, and therefore also include the median grain size^20,22–27^ of the transported sediment.

Published bedload transport datasets from rivers with similar flow conditions, morphologies, and median grain sizes, may show different transport rates, with large variations in the threshold for particles motion^28^, variations that can be up to tenfold^29^ around the mean empirical Shields curve^30–32^. To explain such dispersion, many studies have focused on the role of mixed grain size, hiding effects^33–36^, macro-roughness, channel steepness, or bed roughness relative to channel depth^37^. However, fewer studies have qualitatively studied the influence of pebble shape on bedload transport through its effect on angularity^38,39^, pebble imbrication^34,35,40^, or bed roughness^34,40,41^ (i.e. impact of the *D/K* ratio, where *D* is the diameter of the particles to be moved and *K* is the bed-particle diameter). In environments with smooth-beds (*D* > *K*) and during low to moderate flood events, coarse particles of spherical or ellipsoid shape were observed^42^ to be more likely to experience entrainment and transport than flatter shapes. Conversely, in rough-bed rivers (*D* < *K*), Demir and Walsh^1^ found that displacement of flatter shapes (i.e. discs and blades) seems to be promoted. Overall, selective shape entrainment and travel length both decrease as flood magnitude increases and/or particle size decreases^43^. Whereas these previous studies have emphasized that robust deterministic expression of initial motion should encapsulate the role of particle shape and bed roughness in particle motion modelling^38,39,44,45^, the scarcity of field and experimental data has prevented a quantitative account of this role.

To partially fill this gap, we designed a parametric study based on experiments run in an annular flume (see the method section) in which the displacements (encapsulating onset motion, travel length and rest periods) of artificial pebbles of various shapes and densities were tracked for several hours. Particle shape has been quantified by many different parametrizations^46–52^ expressing angularity, surface roughness, or departure from sphericity. As the latter directly impacts on inertial moments and pivoting angle, we investigated the influence of shape in terms of the departure from sphericity, examining various ellipsoid particle shapes (from plate to blade types).

## Results

**Figure 1:**
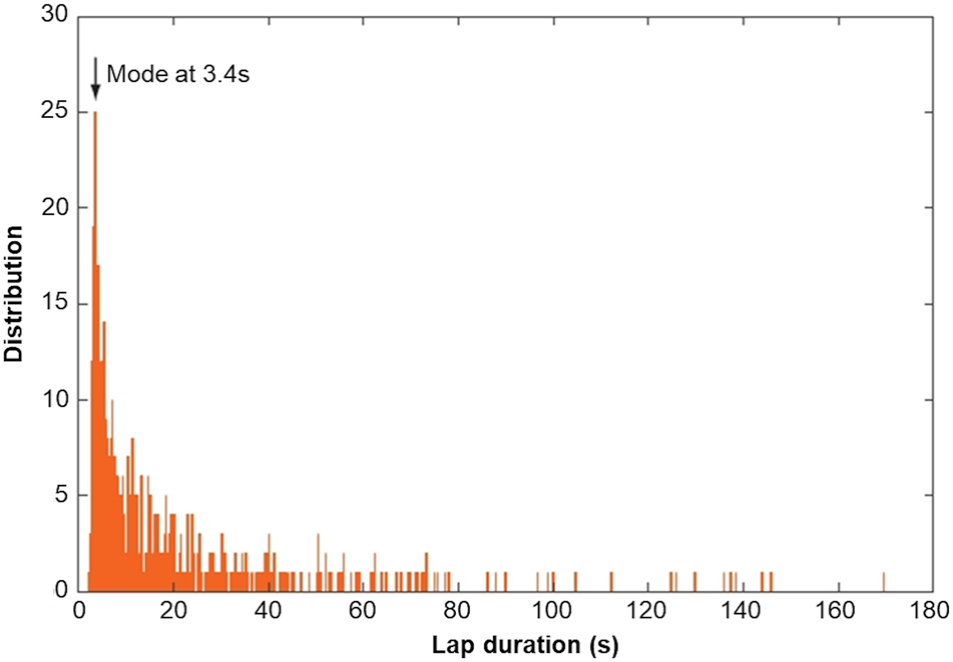
Example of the distribution of lap durations (shape = disc; density = 2.4 g cm^−3^).

**Figure 2:**
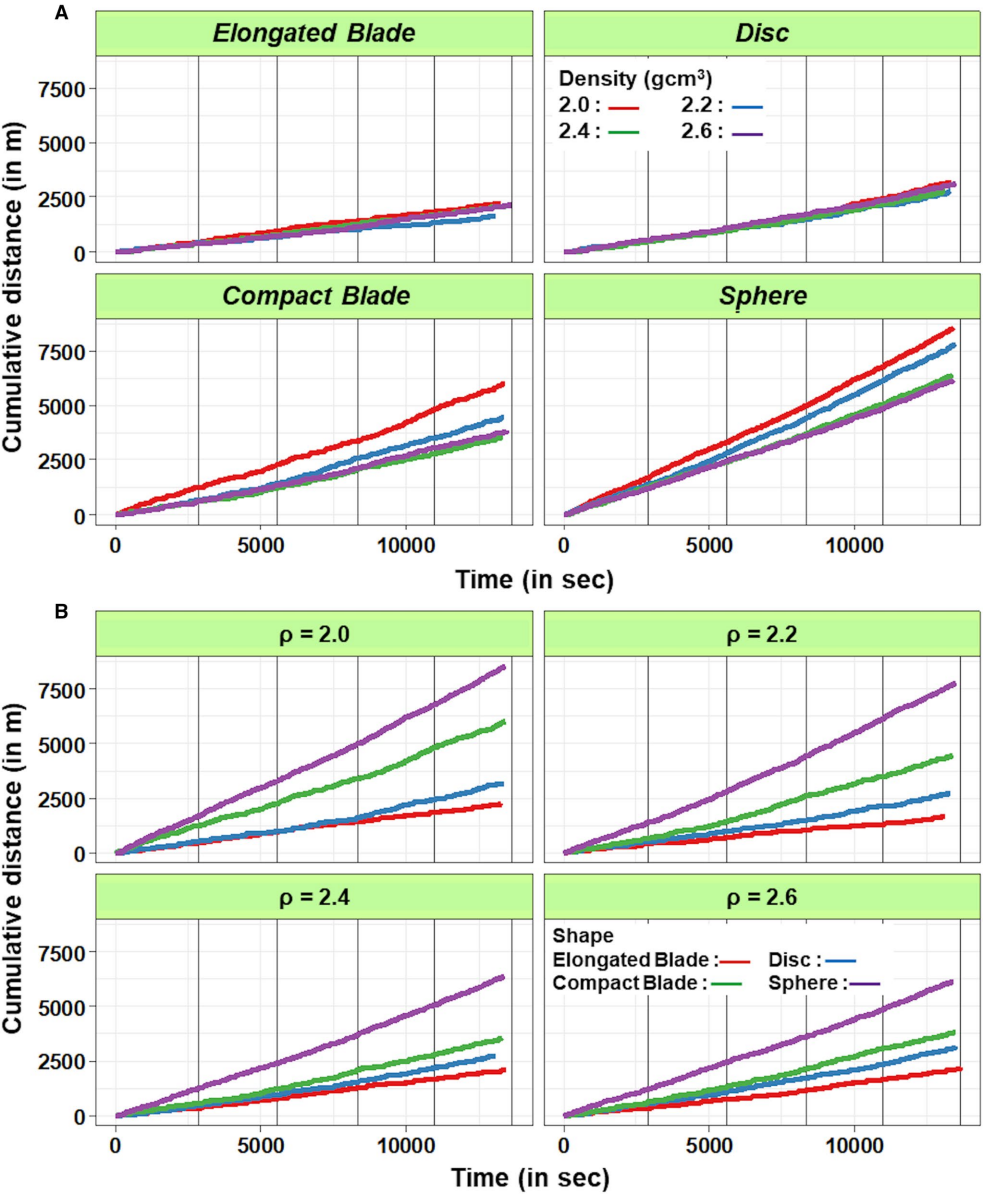
Cumulative travel distances over time according to particle shape (**A**) and density (**B**).

The number of revolutions recorded for the monitored particles ranged between 439 laps for an elongated blade and 2270 laps for a sphere during the same period, ensuring that the lap duration measurements were extracted from large samples. Although the lap durations within the annular flume displayed large variations (from 3 s up to a few minutes; see example in Fig. 1) over the total run duration, the cumulative travel distances of the particles (Fig. 2) displayed a fairly constant slope that permitted the average traveling velocities of the different artificial pebbles to be defined.

The slight increases observed in the slopes of the cumulative distance curves over time for all shapes and densities reflect the progressive augmentation of the particles’ velocities caused by a decrease in the mixing load due to abrasion (relative mass loss of 1.2% per kilometer traveled). As this effect was minor and affected similarly all tagged particles, it was concluded that it had a little impact on the first-order estimates and results of the experiments.

**Figure 3:**
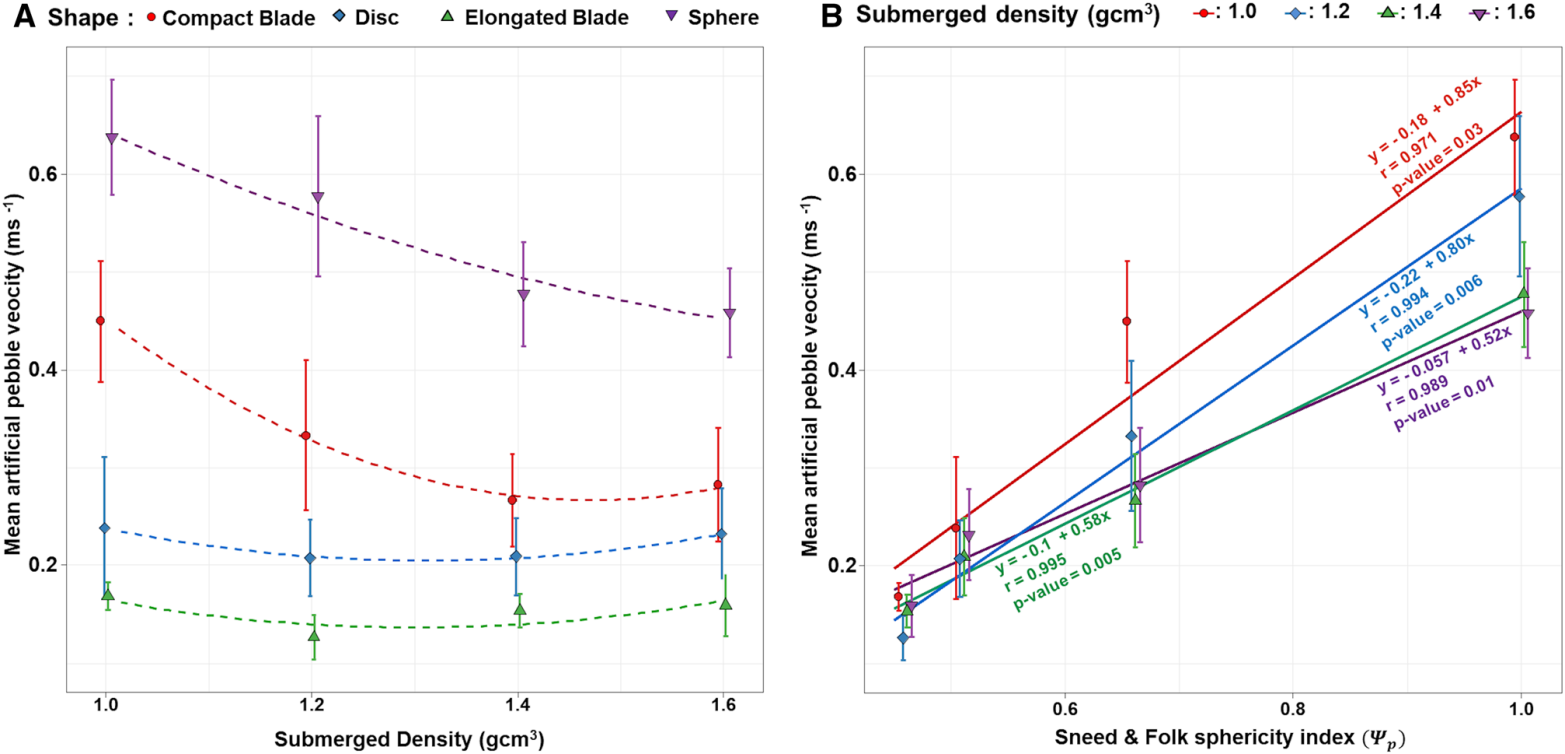
Mean velocity as function of density (**A**) and Sneed and Folk spherical index (**B**).

Both the particle shape and density exhibited significant differences in the cumulative travel length (Fig. 2). The spherical particles traveled the farthest and fastest (mean velocities ranging from 0.44 to 0.60 ms^−1^), with the mean virtual velocity displaying an inverse relationship with density (Fig. 3). The compact blade-shaped particles were the second fastest, exhibiting mean velocities ranging between 0.25 and 0.44 ms^−1^, again displaying an inverse relationship with density, although to a lesser extent than that of the spherical particles. In contrast, the mean virtual velocities of the disc- and elongated blade-shaped particles were minimally influenced by density: the mean velocities were clustered within a narrow range from 0.14 to 0.17 ms^−1^and 0.19 to 0.21 ms^−1^ respectively. Within the density classes, the distances traveled by particles, clearly showed a high variability in relation to their shapes (Figs. 2B and 3A). The experiments clearly indicate that the variability in velocity associated with pebble shape is substantially higher than that associated with particle density (~ 100% compared with ~ 30%).

To explore the influence of particle shape on mobility in a more quantitative way, we used the sphericity index, ${{\varvec{\Psi}}}_{{\varvec{p}}}$ (1), of Sneed and Folks (1958):

1 $$\Psi_{p} = \sqrt[3]{{\frac{S^{2}}{{LI}}}}$$

where *L*, *I*, and *S* are the longest, intermediate, and shortest axes of the pebbles.

The sphericity index ${{\varvec{\Psi}}}_{{\varvec{p}}}$ shows a remarkable positive relationship with the mean traveling velocity (Fig. 3B). Moreover, the mean velocities increased from 0.52 to 0.85 ms^−1^ for decreasing densities from 2.6 to 2.0 g cm^−3^. These results suggest that it is possible to estimate differences in the mean virtual velocities and mobilities of particles according to their sphericity.

**Figure 4:**
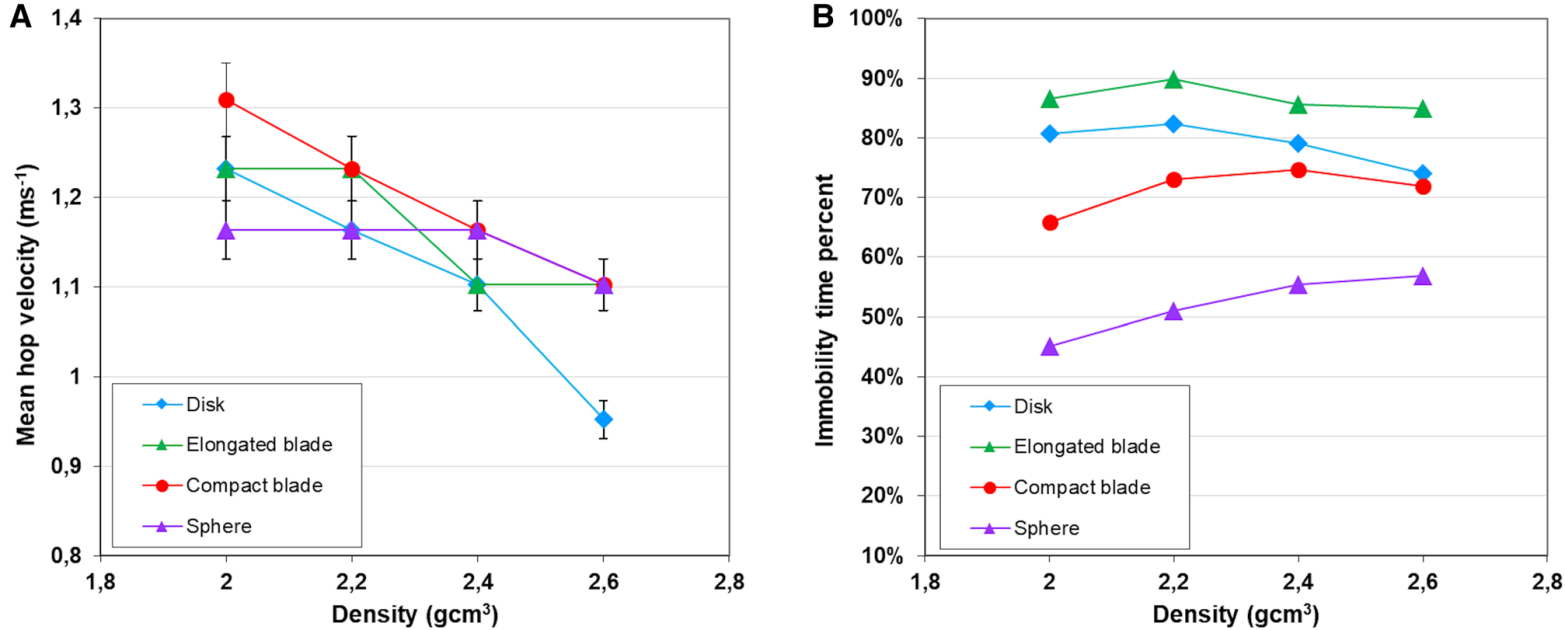
(**A**) Mean hop velocity and (**B**) time fraction of immobility of the 16 different artificial pebbles.

The lap-scaled average travel velocities integrate the duration of motion phases and the resting periods between one phase and the following one. The pebble shape and density can influence the rest and the motion phase differently. The lap duration distributions are characterized by a first peak at around 3 s in all experiments (Fig. 1), which corresponds to a revolution speed of ~ 1.2 ms^−1^. For experimental conditions similar to those used in this study, high speed camera viewing^53^ previously indicated a mean hop velocity of 1.2 ± 0.2 ms^−1^ for pebbles in an annular flume. This modal lap duration of ~ 3 s therefore represents a continuous succession of hops over a full lap, without any resting time. These modal values decrease slightly with increasing density (Fig. 4A), as expected from the larger inertial effect after the pebble is set in motion. More importantly, they are almost independent of the pebble shape, as was also observed in a straight-flume study^54^. This implies that the influence of shape on the mean traveled distance is mostly caused by its influence on the resting time between movements, i.e. on the immobilization conditions and on the threshold for setting pebbles in motion. To illustrate this inference, a simple calculation of the mean resting time fraction, or immobility ratio (I_r_), can be estimated through

2 $${\text{I}}_{r} = \frac{{T - N_{l} t_{m} }}{T},$$

with *T* being the total duration of the runs, *N*_*l*_ the number of achieved flume revolutions during *T*, and *t*_*m*_ the modal lap duration (first mode on the distribution of Fig. 1) corresponding to a continuous succession of hops over a full lap.

Except for spherical pebbles that display a slight increase, the immobility ratio (Fig. 4B) is only weakly or not at all affected by particle density. In contrast, the shape of a pebble deeply impacts on its mobility, with the immobility ratio raging from ~ 50% for the spherical shapes up to ≥ 85% for the elongated blades.

## Discussion

The greater velocity of the spherical and compact-blade-shaped particles that was found in this research compared to the elongated-blade and disc-shaped particles is in good agreement with the literature^1,39,55^, given that the flatness of the flume bottom constitutes a low roughness bed surface, despite clustering of temporary resting pebbles. As most pebble lithologies in rivers show a density close to 2.7 g cm^−3^, which is similar to the highest value used in this study, we expect their mean velocities to be more strongly influenced by their shape than by their density. On a quantitative basis, this supports the claimed need to include a particle shape parameter in the sediment transport equation^34,41,56^.

To do this, we focus on the conditions for setting a particle in motion, starting from the assumption that pebble shape has a major influence on virtual velocity through resting periods. Following Komar and Li's (1986)^41^ description, balancing of the moments of tractive and resisting forces for the critical stress yields:

3 $$\tau_{c} \propto \frac{{l_{W} \Delta \rho gSIL}}{{l_{D} A_{a} }}$$

where *A*_*a*_ is the apparent section exposed to the flow, and *l*_*D*_ and *l*_*w*_ the respective moment arms of the drag force and submerged weight respectively. Assuming that pebbles tend to lie with their *S*-axis vertically oriented, the moment arms of the drag force *l*_*D*_ approximately scales with the *S*-axis. As a pebble can orient either longitudinally or transversally, we use the intermediate variable $\sqrt {LI}$ to account for the apparent section exposed to the flow ($A_{a} \propto S\sqrt {LI}$) and the moment arm of the submerged weight *l*_*w*_. Therefore:

4 $$\tau_{c} \cong k\frac{{\sqrt {LI} \Delta \rho g\sqrt[3]{{\left( {SIL} \right)^{2} }}\tilde{D}}}{{S^{2} \sqrt {LI} }} = k\sqrt[3]{{\frac{{\left( {IL} \right)^{2} }}{{S^{4} }}}}\Delta \rho g\tilde{D} = k\frac{1}{{{\Psi }_{P}^{2} }}\Delta \rho g\tilde{D}$$

where *k* is a function of the particles’ Reynold number considered as a constant, $\tilde{D} = \sqrt[3]{SIL}$, the mean pebble size, and $\Psi_{P} = \sqrt[3]{{\frac{{S^{2} }}{IL}}}$, the Sneed and Folk's index. Here, $\frac{1}{{{\Psi }_{P}^{2} }}$ corresponds more or less to the term tan *ϕ* in Komar and Li (1986): when particle flatness increases (i.e. ${\Psi }_{P}$ decreases), the pivoting angle increases and mobility is reduced. Suppressing the unknown *k*, the threshold can be expressed as:

5 $$\tau_{c} \cong \left( {\frac{\Delta \rho }{{\Delta \rho_{ref} }}} \right)\left( {\frac{{{\Psi }_{{{\varvec{P}}_{{{\varvec{ref}}}} }} }}{{{\Psi }_{{\varvec{P}}} }}} \right)^{2} \tau_{{c_{ref} }}$$

where $\tau_{{c_{ref} }}$ is the critical Shields stress of a reference pebble of similar size.

The non-dimensional critical threshold is expressed as:

6 $$\tau_{c}^{*} \cong \frac{{\tau_{c} }}{{\Delta \rho g\tilde{D}}} = \frac{k}{{{\Psi }_{P}^{2} }} = \left( {\frac{{{\Psi }_{{{\varvec{P}}_{{{\varvec{ref}}}} }} }}{{{\Psi }_{{\varvec{P}}} }}} \right)^{2} \tau^{*}_{{c_{ref} }}$$

where $\tau^{*}_{{c_{ref} }}$ *is* the critical Shields stress of a reference pebble of similar size.

**Figure 5:**
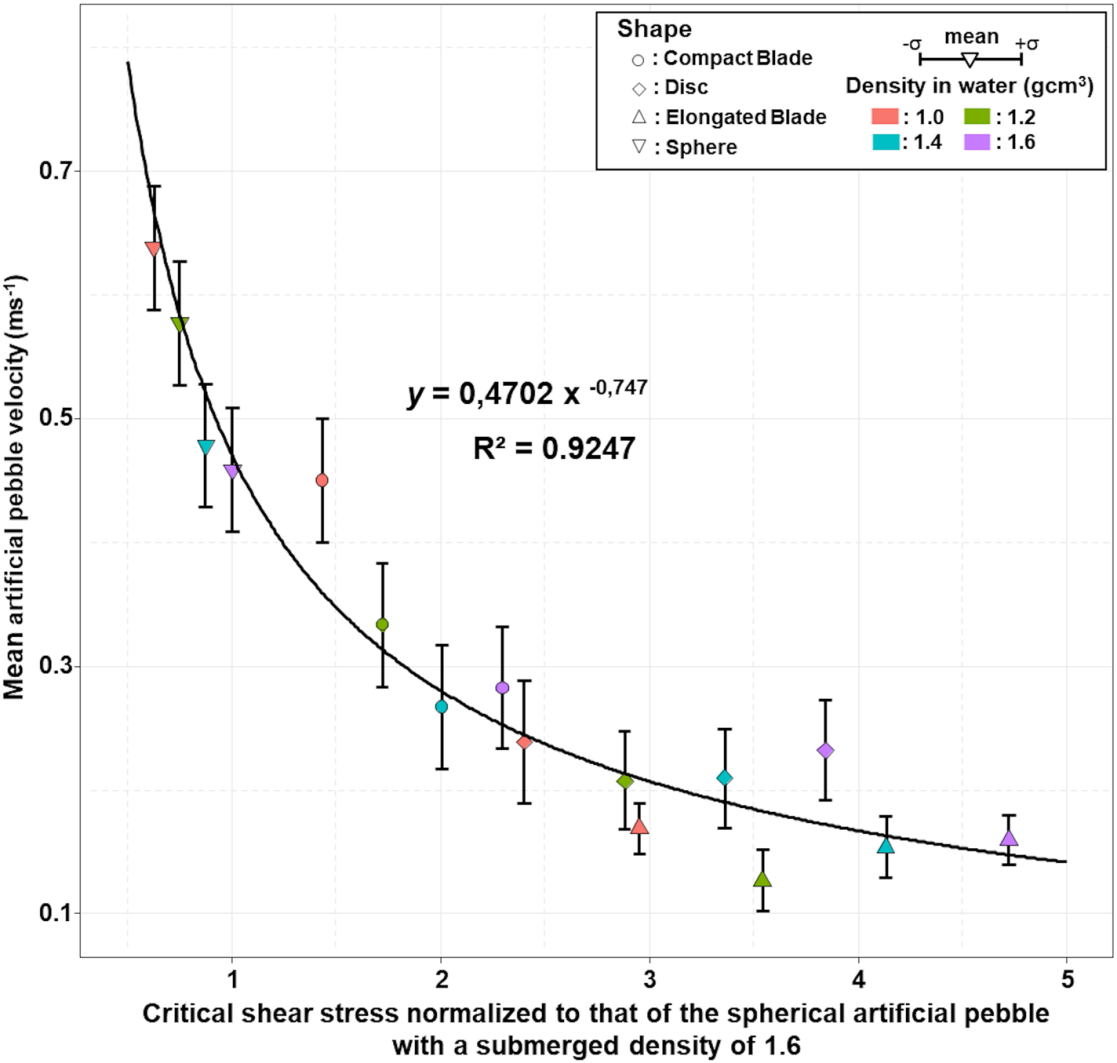
Mean velocity of the 16 artificial pebbles vs. their critical shear stress normalized to that of the spherical artificial pebble with a submerged density of 1.6 $\left( { = \frac{{\tau_{c} }}{{\tau_{{c_{ref} }} }} = \left( {\frac{{\Delta_{\rho } }}{1.6}} \right)\left( {\frac{1}{{\Psi_{P} }}} \right)^{2} } \right)$.

When the mean travel velocity of particles is expressed as a function of the critical stress *τ*_*c*_*,* an inverse correlation between the two variables results (Fig. 5): both an increase of and a decrease of sphericity decrease the ratio of tractive over resistive moments and favor particle immobility.

Most bedload transport capacity formulae are functions of the excess Shields stress and follow two general forms: (1) $\Phi = K(\tau^{*} - \tau_{c}^{*} )^{\upalpha }$, and (2) ${\text{W}}^{*} = \left( {\tau /\tau_{c} } \right)^{\alpha }$, where Φ and ${\text{W}}^{*}$ are two distinct non-dimensional expressions of the bedload transport rate, and α and *K* two constant terms^57^. To account for the role of pebble shape in a transport capacity relationship, one could introduce the modified expression of critical shear stress (Eq. 5) into the formula, or the critical Shields stress (Eq. 6) that includes the Sneed and Folk Index.

To explore this hypothesis, we built on the fractional transport rate model developed for transport of a mixture of grain sizes (e.g. Parker et al., 1982^58^). This choice was motivated by the fact that such a relation already proposes a similarity collapse for heterogeneous sediments, which is the case in our experiments with particles of variable shapes and densities mixed with a natural pebble load. We arbitrarily considered Wilcock and Crowe's (2003)^59^ relation for fractional transport rate, in which the form of the similarity collapse is:

7 $$W_{i}^{*} = 14\left( {1 - \frac{0.894}{{\phi^{0.5} }}} \right)^{4.5} \;{\text{when}}\;\phi = \frac{\tau }{{\tau_{ci} }} \ge 1.35$$

where $\tau$ is the bed shear stress, $\tau_{{{\text{ci}}}}$ the critical shear stress for incipient motion of a specific pebble *i* (more exactly it corresponds to the minimum shear stress required to achieve a small reference transport rate of $W_{i}^{*}$ = 0.002^58^), and $W_{i}^{*}$ the dimensionless transport rate $W_{i}^{*} = \frac{{Rgq_{bi} }}{{F_{i} \left( {\frac{\tau }{\rho }} \right)^{3/2} }}$, with $R_{i} = \frac{{\Delta \rho_{i} }}{\rho }$ being the ratio of the submerged sediment (of type *i*) density to water density, *g* being gravity, *q*_bi_ the volumetric transport rate per unit width of the particle of type *i* (i.e. of similar shape, size, and density), and *F*_*i*_ the proportion of the pebble type being of the class *i.*

Following our simplified analysis of the balance of force momentum, we defined the critical (or reference) shear stress as a function (Eq. 8) of the mean characteristics of the transported sediment load (i.e. mean gravel size *D*_*m*_, mean shape factor $_{Pm}$, and mean density $\Delta \rho_{m}$) according to:

8 $$\tau_{ci} = \left( {\frac{{{\Delta \rho }_{i} }}{{{\Delta \rho }_{m} }}} \right)\left( {\frac{{{\Psi }_{Pm} }}{{{\Psi }_{Pi} }}} \right)^{2} \tau_{cm}$$

with $\tau$_cm_ being the critical shear stress for the mean gravel load. Here, $\tau_{cm} = \Delta \rho_{m} gD_{m } \tau_{c}^{*} \cong 28\,{\text{Pa}}$ considering that $\Delta \rho$ = 2600 kg m^−3^, D_m_ ≈ 5 cm for the mean gravel diameter of the 65 kg of limestone pebbles, and $\tau_{c}^{*} \cong 0.036$^59^.

Within the flume, provided that not all particles are in full motion, the conditions of alluvial rivers prevail, i.e. the sediment flux *q*_*si*_ is equated by the transport capacity *q*_*bi*_. In our experiments, the mass sediment flux per unit width of the pebble class *i* can be expressed from the mean traveling velocity through: $q_{si} = \frac{{F_{i} M}}{{\text{A}}}V_{gi}$, with A being the surface of the flume bottom, *M* the mass of sediment introduced into the flume, and *V*_*gi*_ the mean displacement velocity of particles of type *i*. It follows that a virtual mean velocity can be derived for particle *i* from the above fractional transport rate equation:

9 $$V_{gi} = \frac{{AM\rho_{si} }}{{R_{i} g}}\left( {\frac{\tau }{\rho }} \right)^{3/2} W_{i}^{*} \left( {\frac{\tau }{{\tau_{ci} }}} \right)$$

with $\tau_{ci}$ derived from Eq. (8) and a mean shape factor ${\Psi }_{Pm} = 0.7 \pm 0.08$, which corresponds to the 65 kg of rounded limestone pebbles, most of which have a shape close to that of a compact ellipsoid, and with which our tracked artificial pebbles were mixed.

**Figure 6:**
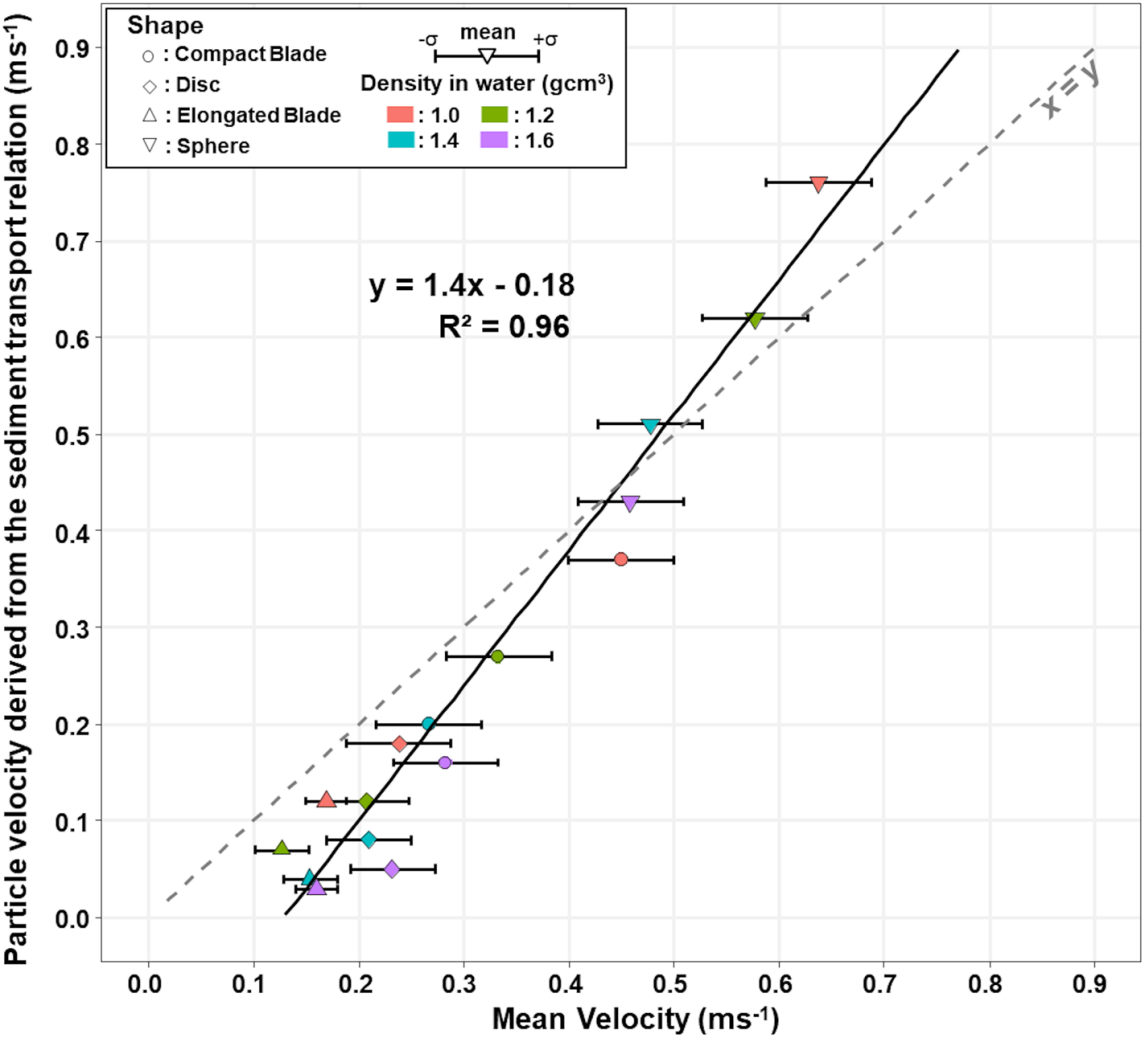
Comparison between the mean measured velocities of particles of various shapes and densities and the theoretical particle velocity derived from a fractional transport rate relation adapted from Wilcock and Crowe’s (2003) relation^59.^

The virtual velocities derived from the bedload transport relation show a well-defined correlation with the measured virtual velocities (Fig. 6). However, the slope of the correlation line is larger than unity, and our modified version of the bedload transport tends to underestimate the observed transport for the densest elongated-blade or disk-shaped pebbles. Despite these slight discrepancies from the observations, these results suggest that the role of pebble shape on bedload transport can be predicted, and that the inclusion of pebble shape characteristics in the modelling of bedload transport offer much promise for improving bedload transport predictions.

In terms of sediment dynamics, pebbles travel in the flume following an alternating pattern of resting and motion periods, as generally observed in a natural stream^8^. We therefore consider that our experiments succeeded in capturing the first order behavior of the bedload, and that the introduction of a shape factor into the critical Shield stress and bedload transport models might be transposed to rivers. However, the experimental conditions are slightly different from those of natural rivers, in particular the use of a monodispersed sediment load and a low-roughness bottom. Additional experiments exploring distinct bottom conditions, grain size distributions or shapes, and using straight channels are probably necessary to strengthen our initial results and resolve the slight discrepancies between the model and observations. Experiments using pebbles with a unique and defined type of shape, instead of a single particle mixed with a large population of pebbles of distinct shapes, would allow, for example, the examination of fluvial transport of predominantly flattened particles such as those resulting from the erosion of schist- or shale-rich lithologies. Similarly, our experiments were conducted with relatively well-rounded limestone pebbles, whereas the upstream reaches of mountain river networks tend to be characterized by particles with more irregular or angular shapes. Although some part work^60^ has suggested no relation between pebble angularity and the resistance to initial movement of the particle, we can hypothesize that significant particle angularity might promote particle imbrication and reduce bedload mobility and transport. Future experiments could thus be designed to explore the influence of angularity on bedload transport capacity. Our experiments presented in this paper, should help to derive a more universal relationship applicable to natural rivers with heterogeneous mixtures of sizes and shapes comprising the bedload. The present study can therefore be considered as a preliminary step towards addressing the role of particle shape in bedload transport. All existing field data sets do not comprise information about grains shape. In order to fully explore these properties, a protocol needs also to be defined for collecting this information in future field campaigns.

## Conclusion

The experiments performed in this research, which are based on innovative tools (artificial pebbles of controlled density containing RFIDs) offer new perspectives for studying sediment transport mechanisms. The comparative analysis of the shape and density of particles on their mobility highlights the crucial influence of particle shape. Furthermore, it also indicates that the sphericity index (***Ψ***_***P***_) of Sneed and Folks (1958)^61^, which correlates with mean velocity, is relevant for including shape parameters in sediment transport formulae. The method developed in this study can be reproduced to investigate how bed roughness (changing *D/K* ratio) and/or a tracer’s grain-size can change the balance between the effects of shape and density on particle velocity. It allows the investigation of whether bed roughness promotes the transport of flat-shaped particles, as reported in the literature, and whether particle density can mitigate this effect. Repeating the experiments with smaller particle sizes (maintaining a constant *D/K* ratio) would also allow investigation of whether size mitigates the influence of shape and density on particle transport.

## Methods

**Figure 7:**
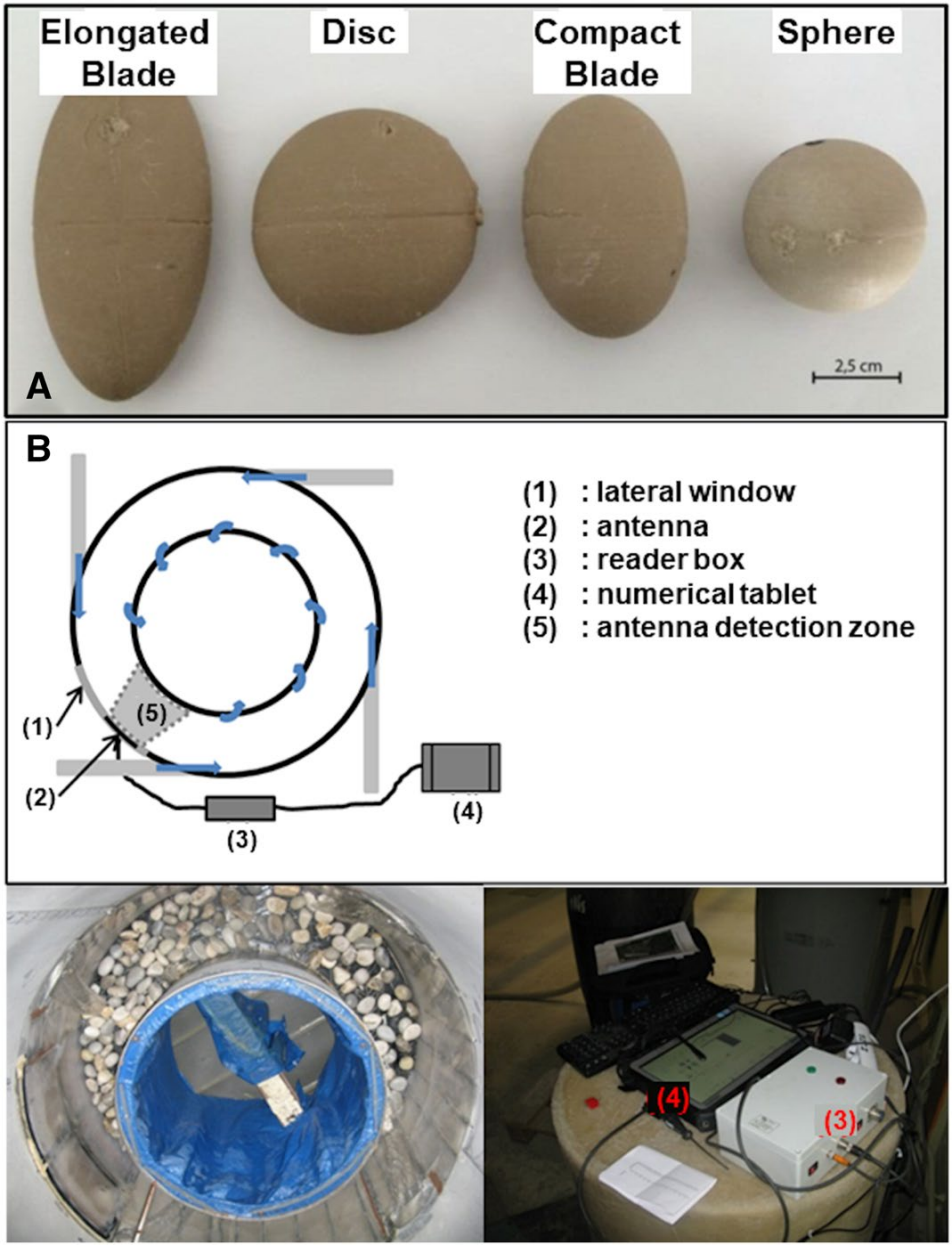
The four particle shapes investigated (**A**) and the annular flume equipped with the RFID system (**B**).

We designed four differently-shaped particle models within the grain-size class of 45–64 mm (5.5–6.0 Ψ-units), with all models having the same volume (i.e. 49.3 cm^3^) but exhibiting differences in the sphericity index^62^ (Fig. 7A; Table 1). After creating silicon molds (RTV 120) for these four models, we manufactured 16 artificial pebbles using a mixture of resin and corundum powder in variable proportions, creating pebbles of four different densities (2.0, 2.2, 2.4, and 2.6 g cm^−3^) for each mold shape^63^. We equipped these artificial pebbles with transponders of Radio Frequency Identification, RFID, (model RI-TRP-WR2B of Texas Instrument, Dallas Texas USA, also known as PIT Tags) to monitor their displacements within an annular flume (Fig. 7B). A detection antenna located on the outside of the flume, along a lateral window, enabled tracking of the number of laps achieved by the RFID-equipped pebbles and the time for each revolution.

**Table 1:** Shape characteristics of the artificial particles tracked in the flume.

SHAPE	*a*-axis (mm) *L*	*b*-axis (mm) *I*	*c*-axis (mm) *S*	Vol. (cm^3^)	Sphericity index (Sneed and Folks, 1958)
Compact Blade	68.1	46	30	49.3	0.66
Sphere	45.5	45.5	45.5	49.3	1
Disc	65	63	23	49.3	0.51
Elongated Blade	97.2	46.1	21	49.3	0.46

In an attempt to reproduce bedload transport conditions, these artificial pebbles were mixed with 65 kg of limestone pebbles of a similar grain-size (i.e. class 45–64 mm) and were run within an annular flume. The external diameter and width of the flume are 1.5 m and 0.3 m respectively, and its outer and inner heights are 1.5 m and 0.6 m respectively^63^. The water circulation that allows the movement of the pebbles at the bottom of the flume is induced by 4 tangential injections at the level of the outer wall of the flume (at a height of 0.6 m). The water then flows over the inner edge of the flume to fall into a tank before being recirculated to the injections by a powerful pump. Pebbles, on the other hand, remain permanently at the bottom of the flume, and travel an average distance of 3.77 m per lap. A set of experiments were run following the designs of previous studies^53,63,64^ for which the sediment dynamics have been characterized^64^, i.e. with a low roughness bottom and a monodispersed grain size distribution. During the experiments, the pump discharge sustaining the fluid injection into the flume was maintained at 240 m^3^h^−1^, which for the introduced sediment mass corresponds^65^ to a shear stress of $\tau$ = 135 Pa at the base of the flume according to Euler theorem applied to the moments, a Shield stress of $\tau^{*}$ = 0.16, a mean transit velocity for pebbles of ≈ 0.4 ms^−1^, and a sediment flux of ~ 24 kg m^−1^ s^−1^. Under these conditions, high speed camera viewing^53^ indicated that the pebbles were transported in the annular flume in a similar manner to that observed^8^ in rivers, with alternating transport phases with rolling and saltation, and resting times caused by temporary blockage and piling of particles.

Each experimental run lasted 45 min. To avoid superpositioning of radio-frequency signals and missed RFID transponder detections^63,66,67^, only the particles (n = 4) with the same density were simultaneously present in the flume, so limiting the number of transponders to four. A total of six runs were achieved for the densities of 2.6 and 2.4 g cm^−3^, and five runs for the densities of 2.0 and 2.2 g cm^−3^. For each artificial pebble, the combined runs provide a long duration of almost 4 h and a large cumulative traveled distance, from which the mean traveled velocity (or virtual velocity as defined by Haschenburger and Church^68^) can be computed and the distributions of the lap times estimated. Finally, in order to investigate the effects of the different shapes and densities on bedload transport, the virtual velocities and lap distributions of the 16 artificial pebbles were compared. The use of an annular flume enabled the acquisition of a relatively long time series compared with typical straight flume experiments^69^ and the sampling of a population of practically uncensored particle trajectories, without the limitations induced by a limited detection window or flume length^70^. This ensured that the ranges of traveled distances, under conditions of continuous movement, were well represented in the experiment. We also made sure that the duration of the experiments (45 min) was much longer than the maximum resting time recorded (~ 5 min). This allowed both avoiding time censorship effects on the distributions of the resting periods and lap times, and increasing the statistical significance of the distributions.
