# Author Correction: Bedload transport in rivers, size matters but so does shape

**DOI:** 10.1038/s41598-022-11278-6

**Published:** 2022-04-26

**Authors:** Mathieu Cassel, Jérôme Lavé, Alain Recking, Jean-René Malavoi, Hervé Piégay

**Affiliations:** 1 University of Lyon, Lab. Environnement Ville Et Société, CNRS UMR 5600, Site ENS de Lyon, 15 Parvis René Descartes, BP 000F-69342, Lyon Cedex 07, France; 2 CRPG-CNRS, 15, Rue Notre Dame des Pauvres, BP 20, 54500 Vandœuvre-lès-Nancy, France; 3 Inrae, UR ETNA, Domaine Universitaire, 2 Rue de la Papeterie, BP 76, 38402 Saint-Martin-d’Hères, France; 4 Département Concessions Eau Environnement Territoires, Electricité De France - EDF/DPIH, Le PRIMAT - 190 Rue Garibaldi, 69003 Lyon, France

Correction to: *Scientific reports* https://doi.org/10.1038/s41598-020-79930-7, published online 12 January 2021

The original version of this Article contained an error in Equation 1, where “S^2^” was incorrectly given as “S”.

$$\Psi_{p} = \sqrt[3]{\frac{S}{LI}}$$

now reads:

$$\Psi_{p} = \sqrt[3]{{\frac{S^{2}}{{LI}}}}$$

The original Article has been corrected.

